# A case of schwannoma at the origin of the right recurrent laryngeal nerve resected under uniportal video‐assisted thoracic surgery

**DOI:** 10.1002/rcr2.1023

**Published:** 2022-08-23

**Authors:** Go Kamimura, Kazuhiro Ueda, Aya Takeda, Koki Maeda, Masaya Aoki, Masami Sato

**Affiliations:** ^1^ Department of General Thoracic Surgery, Graduate School of Medical and Dental Sciences Kagoshima University Kagoshima Japan

**Keywords:** azygos lobe, recurrent laryngeal nerve, schwannoma, uniportal video‐assisted thoracic surgery

## Abstract

Mediastinal neurogenic tumours are mostly derived from sympathetic nerves and intercostal nerves, and vagus nerve‐derived schwannomas are rare. We encountered a tumour originating from the origin of the recurrent laryngeal nerve that was accompanied by the azygos lobe, which made it difficult to approach; it was ultimately able to be removed via uniportal video‐assisted thoracic surgery. This case involved a 63‐year‐old female patient. There were no particular symptoms, but an abnormal chest shadow was noted on an imaging examination. Chest imaging revealed a smooth‐surfaced mass in the upper right mediastinum with the azygos lobe. A diagnosis of schwannoma was made by imaging, and the patient underwent resection via uniportal video‐assisted thoracic surgery. The tumour, which originated from the origin of the right recurrent laryngeal nerve, was sharply removed without causing recurrent laryngeal nerve palsy.

## Graphical abstract

Mediastinal neurogenic tumors are often derived from sympathetic nerves and intercostal nerves, and schwannomas originating from the intrathoracic vagus nerve are rare. In the present case, we performed uniportal video‐assisted thoracic surgery for schwannoma of the origin of the recurrent laryngeal nerve. This case was complicated with the azygos lobe, but recurrent laryngeal nerve palsy was avoided even after surgery, so we report it with some consideration.

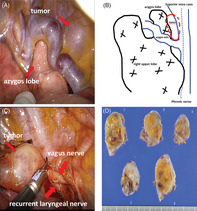

## INTRODUCTION

Mediastinal neurogenic tumours are often derived from sympathetic nerves and intercostal nerves, and schwannomas originating from the intrathoracic vagus nerve are rare. In the present case, we performed uniportal video‐assisted thoracic surgery for schwannoma of the origin of the recurrent laryngeal nerve. This case was complicated with the azygos lobe, but recurrent laryngeal nerve palsy was avoided even after surgery, so we report it with some consideration.

## CASE REPORT

**FIGURE 1:**
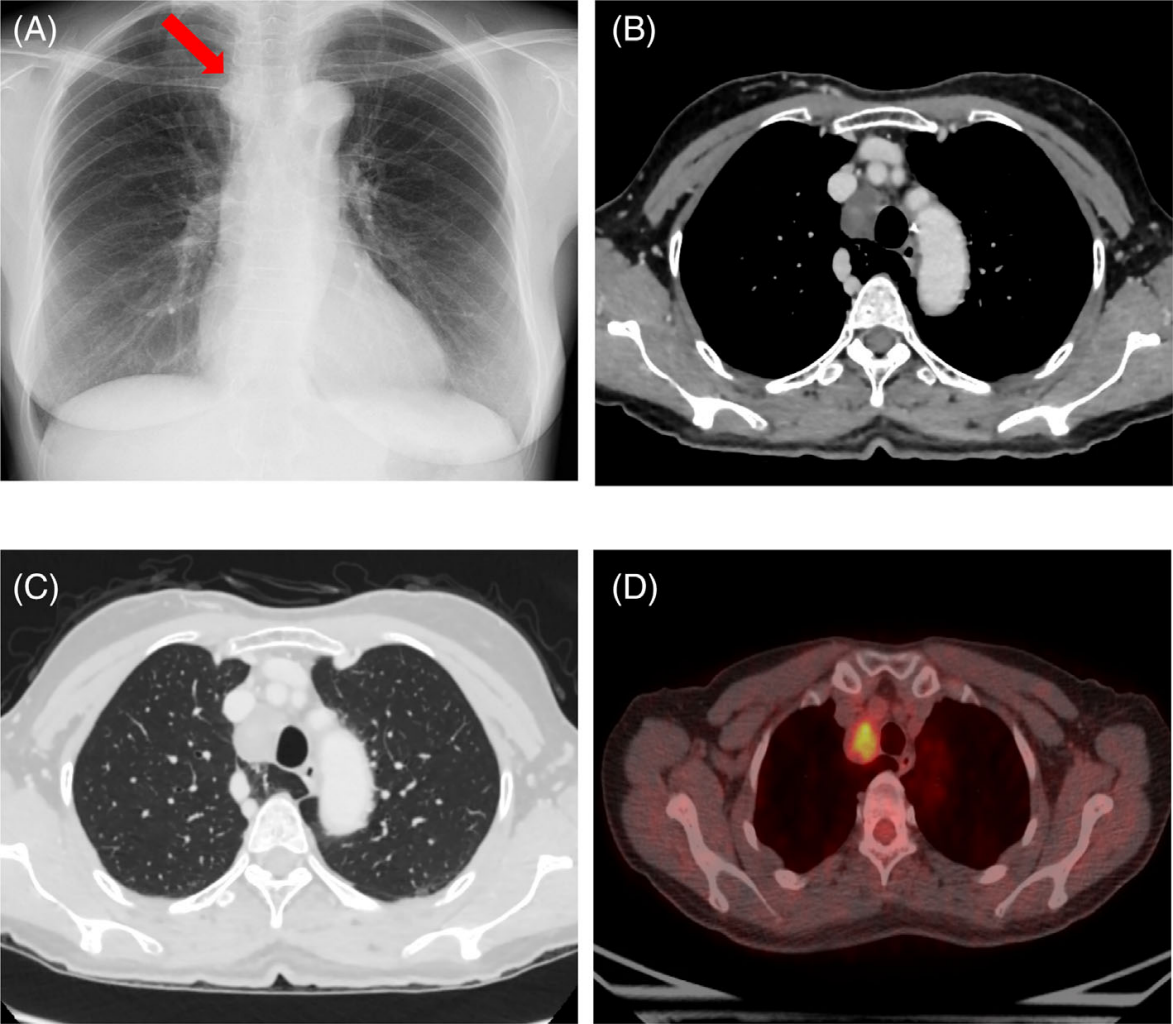
(A) Chest X‐ray revealed a mass in the upper mediastinum. (B) contrast‐enhanced CT revealed a 3‐cm solid lesion with a smooth surface and poor contrast effect slightly excluding the right wall of the trachea, but there was no tendency to infiltrate the surrounding tissue. (C) This case had an azygos lobe near the tumour. (D) ^18^F‐fluorodeoxyglucose positron emission tomography (FDG‐PET) showed a slight uptake in the tumour

The case involved a 63‐year‐old woman with no medical history and no smoking history. Chest X‐ray showed a mass in the upper mediastinum (Figure 1A). Contrast‐enhanced computed tomography (CT) revealed a 3‐cm solid lesion with poor contrast effect in contact with the right tracheal wall (Figure 1B). In addition, the azygos lobe was involved. Based on the location of the tumour, thymoma and neurogenic tumour were suspected, however, in the case of a neurogenic tumour associated with the azygos lobe, it was unclear whether the tumour was of vagal or phrenic nerve origin because the anatomical course of the nerve was unknown (Figure 1C). Contrast‐enhanced magnetic resonance imaging (MRI) revealed a faint contrast effect inside the tumour, however the surface was smooth and no invasion to the surroundings was observed. ^18^F‐fluorodeoxyglucose positron emission tomography (FDG‐PET) showed a slight uptake in the tumour (Figure 1D). Reassessment imaging showed the tumour was enlarging, therefore, surgery was scheduled. The patient was placed in the left lateral decubitus position, and surgery was performed via uniportal video‐assisted thoracoscopic approach with a 4‐cm incision made at the right 5th intercostal space.

**FIGURE 2:**
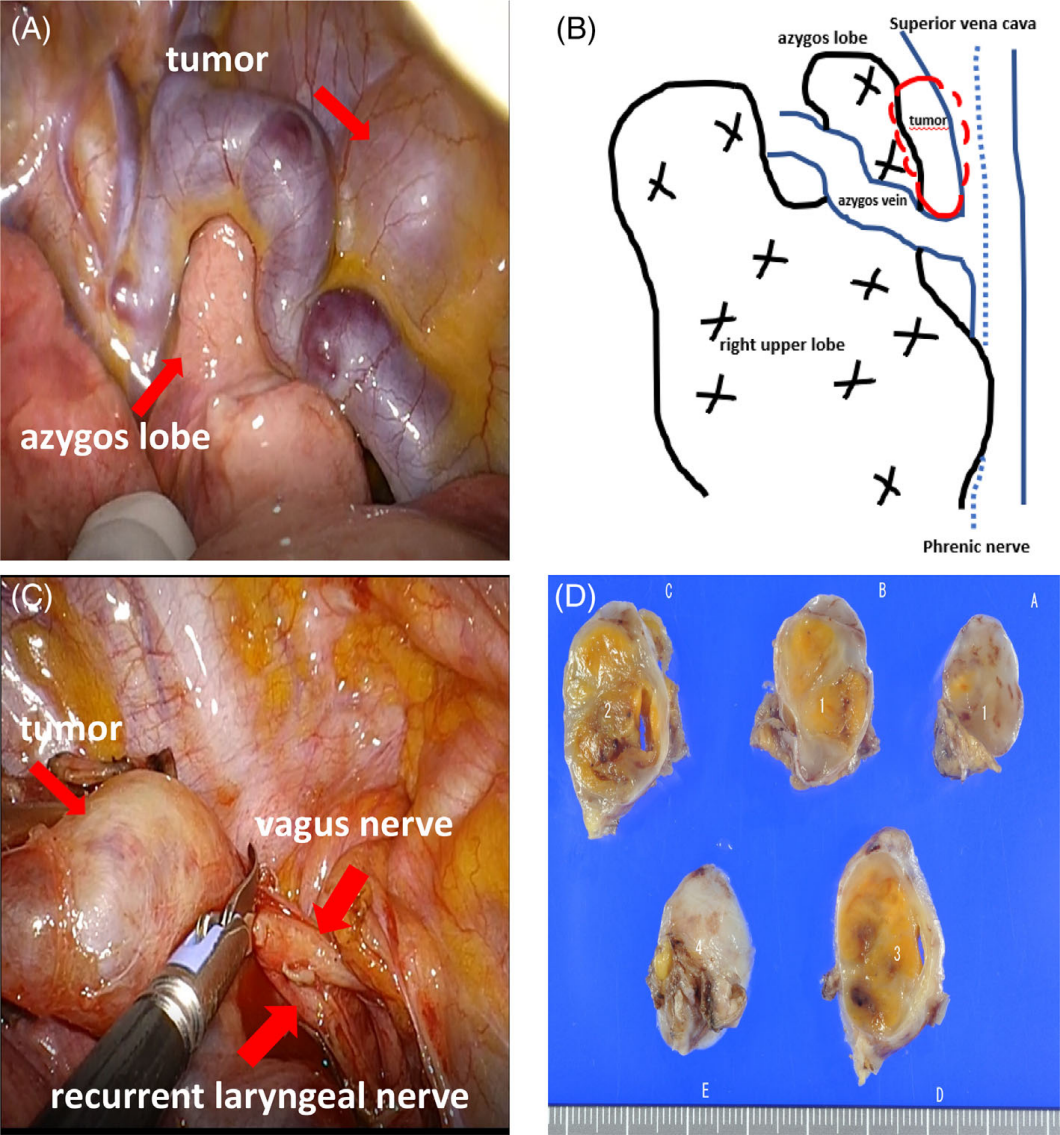
(A) The azygos lobe was observed under the azygos vein, and a tumour was also confirmed near the azygos lobe. (B) The tumour originated from the origin of the recurrent laryngeal nerve. (C) The tumour was sharply resected and removed so as not to damage the recurrent laryngeal nerve. (D) The tumour was diagnosed as schwannoma based on the histopathology

On opening the chest, the azygos lobe was observed under the azygos vein, and a tumour was confirmed near the azygos lobe (Figure 2A). The pleura was incised and dissected, and the tumour originated from the vagus nerve involving the origin of the recurrent laryngeal branch (Figure 2B). The tumour was sharply resected and removed so as not to damage the recurrent laryngeal nerve (Figure 2C).

After the operation, hoarseness was temporarily observed, but the condition gradually improved, and the patient was discharged 7 days after the operation. The pathological findings were a solid tumour of 3 cm (Figure 2D), and spindle‐shaped cells were intertwined in a bundle, showing a palisade arrangement. The tumour cell nucleus was slightly irregular, but there were no mitotic figures, so the patient was diagnosed with schwannoma.

## DISCUSSION

Neurogenic tumours that develop in the thoracic cavity occur primarily in the posterior mediastinum and originate from the intercostal or sympathetic nerves. It is rarely derived from the vagus nerve, and even more rarely at the origin of the recurrent laryngeal nerve.^1^ A preoperative definitive diagnosis is generally difficult for this disease, and surgery is often performed for both the diagnosis and treatment. In addition, this case was complicated by the azygos lobe, and it was necessary to confirm the course of the vagus nerve and phrenic nerve during the operation.

The azygos lobe is not clinically problematic on its own but carries a high risk of complications when performing thoracic surgery. The azygos lobe obstructs the view of vagus nerve confirmation and increases the risk of azygos vein injury.^2^ In addition, it has been reported that 0.6%–3.0% of patients with azygos lobe involvement are associated with congenital heart disease, but preoperative close examinations revealed no abnormalities in this case.^3^

In this case, a vagus nerve tumour originated from the origin of the recurrent nerve was dissected without the use of an electric knife, preserving the recurrent nerve.

Thoracoscopic surgery using multiple ports is generally used for mediastinal tumour surgery; however, in recent years, single‐port thoracoscopic surgery has become widespread, and uniportal video‐assisted thoracic surgery was selected for this case. Regarding the preservation of the vagus nerve, since a long segment of vagus nerve had become the tumour, the vagus nerve below the recurrent laryngeal nerve was excised, and the tumour was removed. Since schwannoma rarely regrows, even after subtotal resection, and recurrence is rare, reduction surgery is reportedly also acceptable when paralysis due to nerve resection has a significant impact on daily life.^4^ Since schwannomas are asymptomatic and rarely malignant, even if they grow slowly, Schulze et al. also suggested partial resection to preserve the vocal cord function.^5^

We experienced a case in which a schwannoma at the origin of the recurrent laryngeal nerve with an azygos lobe was resected. Uniportal video‐assisted thoracic surgery was effective as a minimally invasive and safe method.

## AUTHOR CONTRIBUTION

Go Kamimura is the first author and Kazuhiro Ueda is the corresponding author of this manuscript. Aya Takeda, Koki Maeda and Masaya Aoki participated in the operation of this case. Masami Sato supervised the operation and the editing of the manuscript, Go Kamimura and Kazuhiro Ueda drafted the manuscript, and all authors read and approved the final manuscript.

## CONFLICT OF INTEREST

None declared.

## ETHICS STATEMENT

The authors declare that appropriate written informed consent was obtained for the publication of this manuscript and accompanying images.

## Data Availability

The data that support the findings of this study are available from the corresponding author upon reasonable request.
